# Personalized Robotic-Assisted Total Knee Arthroplasty with Anatomo-Functional Implant Positioning for Varus Knees: A Minimum Follow-Up of 5 Years

**DOI:** 10.3390/jpm15120617

**Published:** 2025-12-10

**Authors:** Zakee Azmi, Aymen Alqazzaz, Cécile Batailler, Sébastien Parratte

**Affiliations:** 1 International Knee and Joint Centre, Hazza Bin Zayed St., Abu Dhabi P.O. Box 46705, United Arab Emirates; 2 Department of Orthopedic Surgery, University of Pennsylvania Health System, Philadelphia, PA 19104, USA; 3 Orthopaedic Surgery and Sports Medicine Department, FIFA Medical Center of Excellence, Croix-Rousse Hospital, Lyon University Hospital, 69004 Lyon, France; 4 IFSTTAR, LBMC UMR_T9406, Claude Bernard Lyon 1 University, 69622 Lyon, France

**Keywords:** total knee arthroplasty, ROSA personalized alignment, robotic-assisted surgery, patellar tracking, ligament balancing, lateral gap laxity

## Abstract

**Background/Objectives**: Some personalized alignment (PA) concepts have been described with symmetrical gaps in extension and flexion. However, laxity in native knees was significantly greater laterally than medially with respect to both extension and flexion. We hypothesized that a personalized alignment can restore the native knee alignment, keep a satisfying patellar tracking, and obtain physiological ligament balancing, that is, a symmetric gap in extension and an asymmetric gap in flexion. We aimed to assess: (1) the postoperative alignment of TKA and postoperative patellar tracking (primary outcome); (2) the ligament balancing at the end of the surgery; and (3) clinical outcomes and complication rates. **Methods**: In this single-center, retrospective case series, we evaluated 45 patients in a consecutive series who underwent robotic-assisted primary TKA using PA between January and September 2020 with a minimum follow-up of 5 years. Complication was defined as grade ≥3 according to the Clavien-Dindo classification. Data assessed were: TKA alignment and implant positioning on postoperative radiographs, patellar tracking on the merchant view, and ligament balancing in extension and flexion upon completion of surgery. **Results**: Mean follow-up was 62.1 ± 2.5 months. The postoperative mean HKA angle was 177.4° ± 2.2. The medial distal femoral angle was restored (91.1° ± 1.5 postoperatively versus 91.3° ± 2). A total of four TKAs had a patellar tilt superior to 5° (8.9%). No significant difference was found in the medial gap laxity—both in extension and in flexion—and the lateral gap laxity in extension. The lateral gap laxity in flexion was significantly higher than extension or medial gap laxity (+2.9 mm). One patient was readmitted for delayed wound healing. Average improvements in Knee Society knee and function scores were 55.86 and 51.84 points, respectively. **Conclusions**: This personalized alignment technique using anatomo-functional implant positioning allowed restoration of native knee alignment with a “safe zone” (3° varus/valgus) for the tibial implant, maintained satisfying patellar tracking, and restituted the asymmetrical gap laxity in flexion with a higher laxity in the lateral compartment. Being the longest system-specific study to date, the results are encouraging at 5 years with no major complications. However, longer follow-up will be required to confirm the use of this technique.

## 1. Introduction

Traditionally in total knee arthroplasty (TKA), mechanical alignment (MA) concepts were used to achieve neutral alignment with femoral and tibial components positioned at 90° to the mechanical axis [1,2,3]. Central to this alignment philosophy lies the notion of reducing wear and risk of loosening by equalizing load across the implant rather than restoration of normal knee kinematics, and it remains the gold standard technique in TKA. This concept automatically led to changes in the joint line orientation by removing some femoral valgus bone and some of the tibial varus. Furthermore, to achieve proper ligament balance, described as symmetrical gaps in extension, ligament releases on the medial side are required, with potential unintended consequences in terms of knee stability in flexion leading to patient dissatisfaction [4]. To obtain symmetrical gaps in flexion, a systematic 3 degrees of lateral rotation of the femur was applied for most of the cases. During the last decade, new alignment paradigms defined as personalized alignments (PAs) have been described with the goal of restoring a more anatomical alignment and avoiding ligament release [5,6,7,8,9,10,11]. To personalize implant positioning and the knee alignment, different concepts of personalized alignment (PA) have been described: kinematic, restricted kinematic, inverse kinematic, and functional [12,13,14,15,16,17]. For PA techniques reported in the literature, the ligament balancing is briefly described and follows the traditional assumption that a symmetrical gap in extension and in flexion should be achieved. In fact, a common historical assumption was that better results would be achieved if symmetrical gaps in both extension and in flexion are restored in TKA. This assumption is classically admitted even if, in native knees, the lateral laxity is significantly greater than the medial laxity, particularly in flexion [18,19,20,21]. The combination between PA and a ligament balancing closer to the one observed in the native knee has been described in cruciate-retaining TKA, although choosing the exact goal in TKA remains challenging using either conventional instrumentation or assistive technologies such as robotics [22]. It is noteworthy that due to PCL resection, the alteration of lateral restraint is thought to be a concern in PS-TKA. One way to approach the challenge of choosing the ideal target in TKA is to think about the functional goals for the patients after TKA. These goals are simple and as follows. First, to obtain great stability during walking activities, particularly during the heel strike phase of the gait, and avoid any varus thrust, for which stable and symmetrical medial and lateral gaps in extension without any lateral opening are prerequisites [23]. The second goal is to maintain a stable knee throughout the arc of knee flexion, particularly on the medial compartment, to avoid any mid-flexion instability. The third goal is to obtain a pain-free range of knee flexion, which requires an adapted femoral roll-back without increasing the tension of the lateral retinaculum [24]. To achieve these functional goals, we proposed the concept of anatomo-functional implant positioning (AFIP), which involve not only the alignment (angular positioning of the implants) but also the restoration of the shapes and dimensions of the distal femur and the proximal tibia, including the joint line orientation, the femoral off-set, a proper patello-femoral tracking, and a physiological ligament balance.

We hypothesized that using this AFIP technique in varus knees will allow the restoration of not just the native knee alignment but also preserve the joint line orientation, the femoral offset, and the patellar tracking as well as a physiological ligament balancing in extension (symmetric gap) and flexion (asymmetric gap). Valgus knees were not included owing to their surgical complexity.

Therefore, after the description of the surgical technique of AFIP using a robotic arm, we aimed to assess: (1) the postoperative alignment of posterior-stabilized TKA on radiographs as measured by limb alignment, femoral, and tibial mechanical axis, tibial slope, the postoperative joint line orientation, the femoral offset, and the patellar tracking as measured using standardized radiographic measures; (2) the ligament balancing in extension and flexion after the TKA implantation measured using the robotic-assisted system; and (3) clinical outcomes and rate of complications within the five-year follow-up period.

Presently, this study is the longest system-specific report of survivorship using AFIP.

## 2. Materials and Methods

### 2.1. Patients

We retrospectively reviewed a consecutive series of patients who underwent primary posterior-stabilized TKA with an AFIP alignment in our institution between 1 January 2020 and 30 September 2020. By this point, the senior author, having experience with the robotic arm, had surpassed the proficiency phases for the learning curve previously reported for this system [25,26]. During this time, the surgical technique was determined based on the availability of robotic assistance and absence of any contraindication for its use. End stage knee osteoarthritis in at least one of the three knee compartments was the indication for surgery. Exclusion criteria included preoperative genu valgus defined as HKA angle (hip knee ankle) superior to 180°, preoperative extra-articular deformity superior to 10°, patellar maltracking with preoperative patellar tilt superior to 20° or patellar subluxation, and previous femoral or tibial osteotomy. These choices were made to design a homogeneous group that allows for uniform application of the surgical technique. Valgus knees and deformities >10° are known to be challenging cases; furthermore, their relative rarity compromises the homogeneity of the group [27].

**Figure 1:**
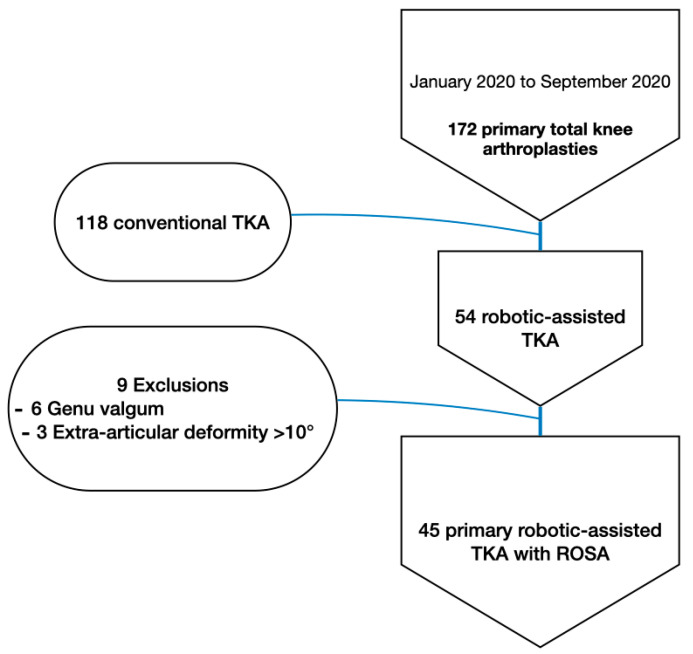
Flow diagram of patient selection.

Out of the 172 primary TKA performed during the study period, 45 robotic-assisted TKAs met the inclusion criteria and were included in the series (Figure 1). No patients were lost to follow-up. In prioritizing prompt reporting of results in order to rapidly assess the need for technique modification—this is the longest reported system-specific survivorship—we opted not to include a control group with conventional TKA.

The mean age was 60.8 ± 7.5 years. A total of 64.4% were female patients (*n* = 29), and 53.3% of operated knees were on the right side (*n* = 24). The mean body mass index (BMI) was 33.5 ± 5.4 kg/m^2^ (Table 1).

### 2.2. Preoperative Knee Analysis

**Figure 2:**
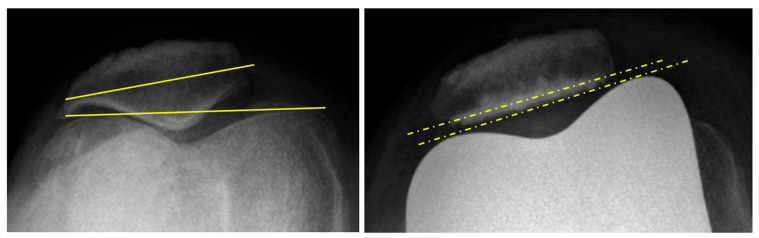
Merchant view preoperatively and postoperatively, with a preoperative patellar tilt of 8.7° and no postoperative patellar tilt.

The aim was to recreate the patient anatomy, avoid ligament release, and to restore both the joint line orientation [10] and the limb alignment. Prior to surgery, we ensured the absence of major extra-articular deformity on the preoperative radiographs. This technique is not intended to correct an extra-articular deformity greater than 10° inside the joint. Standardized radiographic measurements were performed on X-rays taken at the same place: HKA angle (hip knee ankle), mechanical medial distal femoral angle (mMDFA), medial proximal tibial angle (MPTA), tibial slope, joint line convergence angle (JLCA) [6], and joint line orientation (JLO) [28]. Subsequently, patellar tracking is then assessed (Figure 2), and any major abnormal patellar tilt (more than 20°) is identified.

### 2.3. Surgical Technique of Anatomo-Functional Implant Positioning (AFIP)

The same cemented morphometric posterior-stabilized implant (Persona^®^ Knee System, Zimmer Biomet, Warsaw, IN, USA) was used in all patients. All implantations were performed with the ROSA^®^ Knee System using the imageless mode (version 1.2) [29] (Zimmer Biomet, Montreal, QC, Canada). The surgical approach was a tourniquet-less medial subvastus approach without any medial or lateral release and a limited and conservative exposure of the medial tibial plateau. After the robotic set-up and the positioning of the femoral (inside the incision) and tibial trackers (outside the incision), the first step was to assess the frontal laxity in extension and at 90° of knee flexion before the section of the cruciate ligaments. This surgical technique is used as “femur driven” for practical reasons (better access to the tibia without tibial anterior subluxation); therefore, the entire femoral preparation was performed before the tibial cut. Femoral planning was performed to restore the alignment of the femur in a kinematic manner (compensating for the wear); the implant is fundamentally intended to recreate the native anatomy prior to the development of the osteoarthritis vis-à-vis joint line, femoral axis, and rotation [30]. The femur was positioned at the proper femoral mechanical frontal axis to be able to obtain similar resection amounts for both distal condyles that are equal to the implant thickness minus the wear for the worn medial side. No additional femoral rotation was used during the posterior resection that is aligned to the posterior condylar axis. Implant size was then chosen (considering an anterior cut flush to the anterior cortex and 2 degrees of femoral flexion) to ideally restore the posterior condylar offset without anterior overstuffing and avoiding femoral notching [26]. The planning for the tibial cut was then performed using anatomical principle for both the frontal plane and the slope. The goal was to use the lateral unworn compartment to set up the thickness of resection and use it on the lateral side. For the medial side, an estimation of the distance between the deepest part of the tibial plateau and the line of insertion of the fibers of the deep-MCL (which is usually the line just below the medial osteophytes) was performed and taken to be the thickness of the resection. The amount of tibial varus was dialed in on the planning screen to reach these values with a maximum limit of 4 degrees. With respect to the slope, values were intended to mirror the naturally occurring slope up to 9 degrees. Cuts were performed using the robotic arm; post-final verification of the planning, starting with the distal femoral cut, was followed by the positioning of the femoral cutting block using the anatomical principles [31], abiding by the soft tissue envelope of the knee. Resection was limited medially using the line of insertion of the deep MCL [32]. Laterally, the limit of resection was just above the fibers of the anterolateral ligament inserted on the Gerdy tubercle.

Restrictive kinematic principles were used to obtain the desired ligament balancing [33]. The tibial component position was aligned to the tibial mechanical axis up to 3° of varus. The tibial slope was planned to reproduce the native slope of the patient, with a limit of 10°—this cutoff was placed after deciding there would be no clinical utility in reproducing the severe slopes that are prevalent in this geographical region [34]. The robotic-assisted system allowed us to plan this resection with an accuracy of 0.5°, avoiding potential outliers. Bone landmarks can also be helpful and confirm the planning. The tibial landmarks for the tibial resection were the following: (1) Just above the insertion of the deep medial collateral ligament (MCL) in the medial compartment. This landmark usually corresponded to the level just below the medial osteophyte. (2) In the lateral compartment, the limit of resection was just above the fibers of the anterolateral ligament inserted on the Gerdy tubercle. These landmarks corresponded to the limit of the soft tissue shell of the knee joint [32]. For these patients, the gap laxity of the robotic-assisted system was not used to determine the bone resections. These bone resections (thickness and axis) were planned only with the preoperative radiographs and the wear assessment. The ligament balancing was assessed manually to obtain a perfect symmetrical gap in extension (aiming for 1 mm of laxity both for the medial and lateral side of the knee) and an asymmetric gap in flexion (a perfectly stable medial compartment all along the arc of knee flexion; a moderate laxity from 2 to 6 mm in the lateral compartment). The patella was resurfaced when indicated [35] using conventional instrumentation without the use of robotic assistance [36].

A standardized postoperative protocol was applied for all patients with early active range of motion and full weight bearing.

### 2.4. Data Assessment

The data assessment was realized at the first postoperative consultation for the radiographs (1 month) and intraoperatively for the ligament balancing. The radiographic assessment included: anteroposterior view, lateral view, patellar axial view, and standing long-leg radiograph. Axial views were performed using the Merchant method [37]. The standard radiographic measurements were performed: HKA angle, mMDFA, MPTA, JLO, and tibial slope. The JLO was the angle formed between a line parallel to the floor and the tangential to the medial and lateral tibial plateau [28]. Positive values represent a lateral open angle and negative values a medial open angle. Patellar tilt was measured as the angle between the patellar cut surface and the tangent to the anterior border of femoral condyles (Figure 2) [38]. Angles opening medially received a positive value. The patellar translation was measured by the distance between the middle of the prosthetic trochlear groove and the middle of the prosthetic implant of the patella. The threshold was 5° for patellar tilt and 5 mm for patellar translation [39,40]. Radiological measurements were performed twice by one independent reviewer for all measurements to assess the reliability of each measure. The implants positioning on the postoperative radiographs were compared with the planned axis of the bone cuts to evaluate the accuracy of the robotic-assisted system.

**Figure 3:**
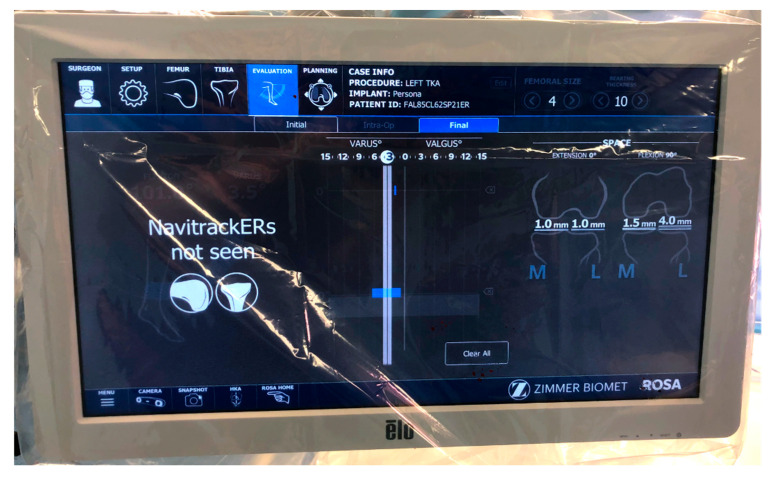
Screening of the ligament balancing assessment after the implantation of the definitive TKA, showing a symmetric gap in extension and an asymmetric gap in flexion.

The ligament balancing was recorded using the robotic-assisted system after TKA implantation with the extensor mechanism reduced (Figure 3). This system allowed measurement of gaps in millimeters in full extension and at 90° of flexion. For each gap, tension was applied manually until resistance was met, and further distraction would have required application of a considerably greater force.

### 2.5. Clinical Outcome

The Knee Society Score (KSS) was used to assess clinical outcome. These scores were collected by patient-filled questionnaires and the operating surgeon. Preoperative scores were collected at the final consultation prior to surgery. Postoperative scores were calculated biannually during consultations. A comparison of the difference between preoperative and postoperative scores at the end of the follow-up period was used as an indicator for clinical improvement.

### 2.6. Complications

We assessed the incidence of serious complications both in the immediate postoperative course and within the specified follow-up period. Serious complications were defined as 3a or greater as per the Clavien-Dindo classification, i.e., events requiring surgical intervention [41].

### 2.7. Statistical Analysis

Statistical analysis was performed using XL STAT software (version 2024.3). Categorical outcomes were compared using Fisher’s exact test and the chi-squared test. The Shapiro–Wilk test was used for normality testing, and a resulting *p*-value of *p* > 0.05 was used to assume data was normally distributed. Normally distributed continuous variables were compared using Student’s *t*-test. Continuous variables were averaged and reported with standard deviations. A *p*-value < 0.05 was considered statistically significant for all analyses.

Intra-rater reliability was assessed for all continuous radiological measurements by repeating them on all 45 radiographs. The reliability was calculated using the intraclass correlation coefficient (ICC) based on a two-way mixed-effects model and absolute agreement for single measures (ICC_3,1_). The strength of the ICC value was interpreted as follows: <0.50 = poor reliability, 0.50–0.75—moderate reliability, 0.75–0.90 = good reliability, and >0.90 = excellent reliability [42]. The radiographic measurements showed good to excellent intra-rater reliability, as depicted in Table 2.

### 2.8. Ethical Approval

All procedures were performed in accordance with the ethical standards of the institutional and/or national research committee, the 1964 Helsinki Declaration, and its later amendments, or comparable ethical standards.

## 3. Results

The mean follow-up time for the series was 62.1 ± 2.5 months, with all patients meeting the minimum required follow-up threshold of 5 years.

### 3.1. Alignment

**Figure 4:**
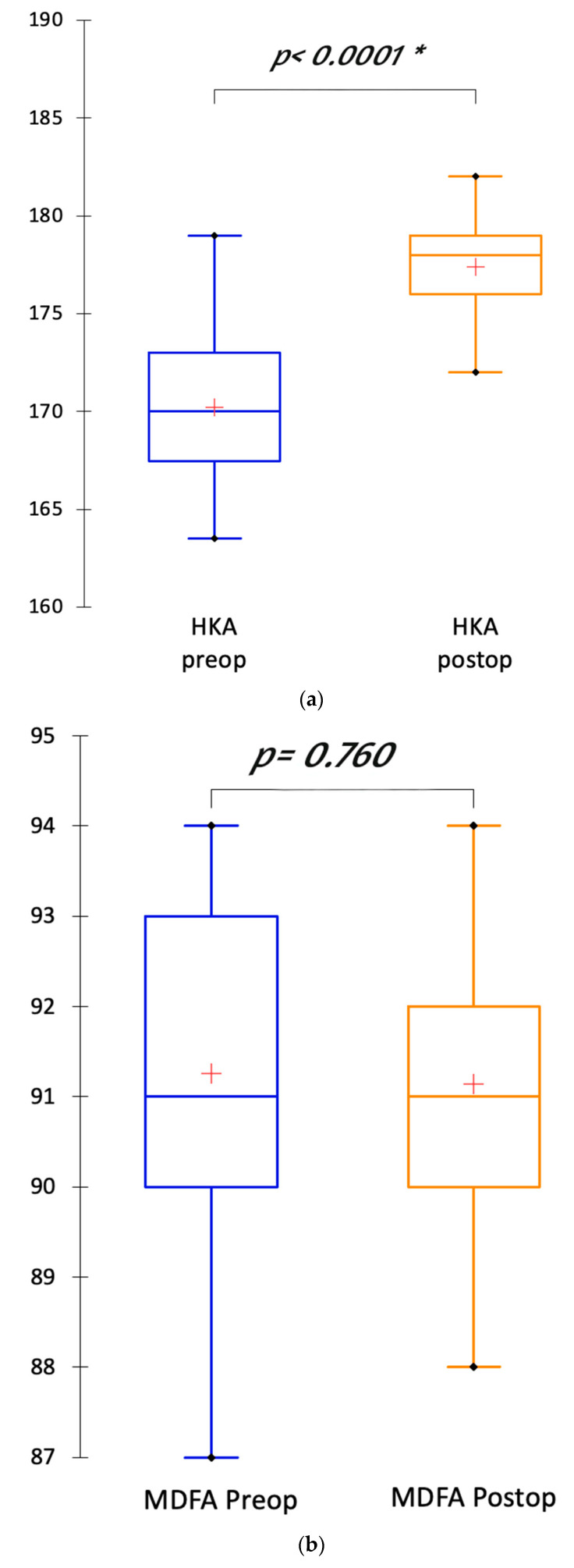

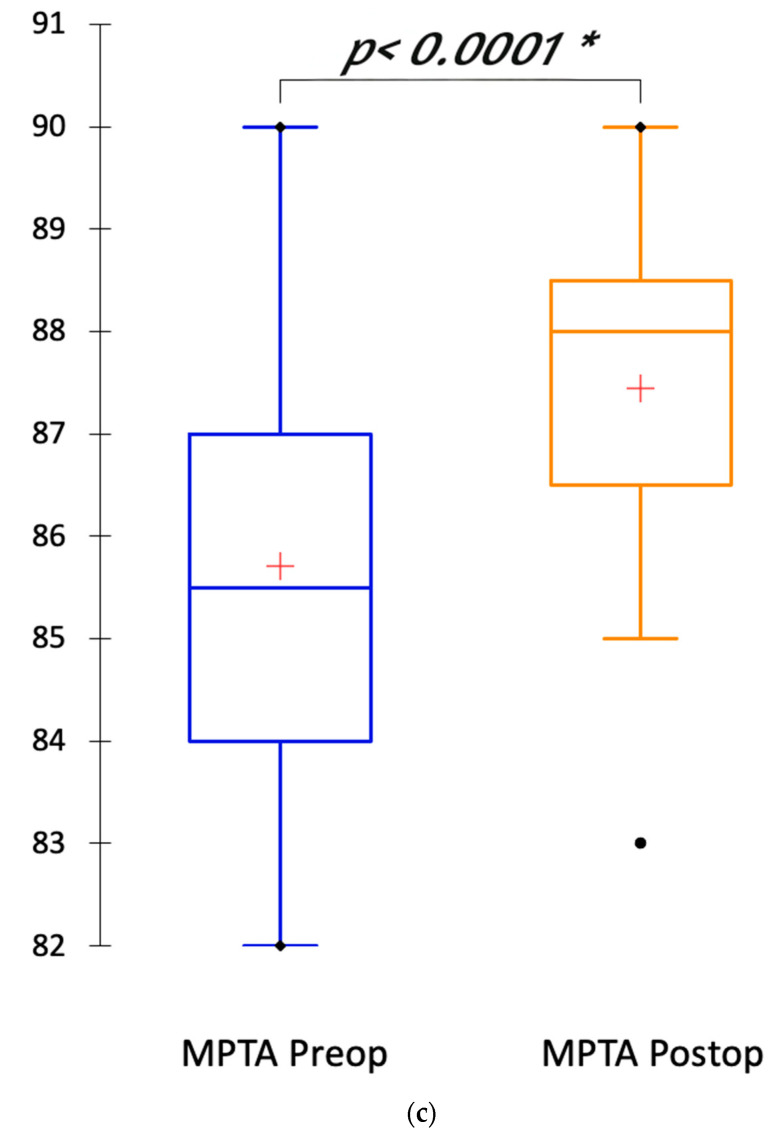
(**a**) Boxplots depicting the transformation of the hip knee ankle (HKA) angle between preoperative and postoperative radiographs; (**b**) Boxplots depicting the restoration of the mMDFA postoperatively compared with preoperative mMDFA; (**c**) Boxplots depicting the partial correction of the MPTA between preoperative and postoperative radiographs. “*” indicates threshold for statistical significance is met. Symbols: cross indicates mean value; dot indicates outlier value.

Limb alignment and components’ positioning data are summarized in Table 3 and Figure 4.

**Figure 5:**
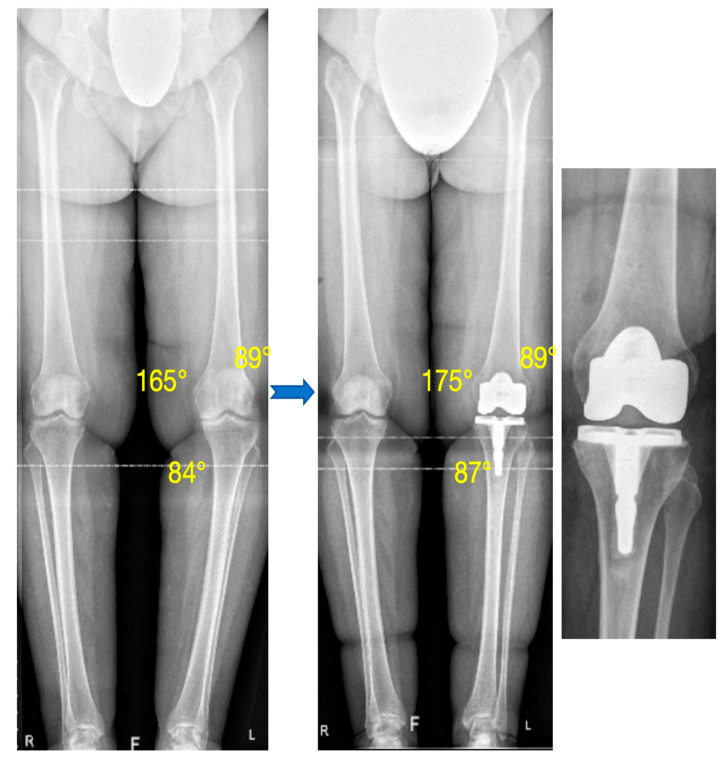
Preoperative and postoperative standing long-leg radiograph. The preoperative varus (preoperatively, HKA: 165°, mMDFA: 89°, MPTA: 84°) was partially corrected with the TKA with restoration of the femur and partial correction of the tibial varus (postoperatively, HKA: 175°, mMDFA: 89°, MPTA: 87°).

The mean postoperative HKA angle was 177.4° ± 2.2. It was significantly less varus than the preoperative HKA angle (170.2° ± 3.9; *p* < 0.0001). This HKA correction was due to the partial correction of the MPTA (87.4° ± 1.8 postoperatively versus 85.7° ± 2.1; *p* < 0.0001) and the wear compensation (JLCA = 6.2° preoperatively). The mMDFA was not significantly different in the postoperative radiograph compared with preoperative radiograph (91.1° ± 1.5 postoperatively versus 91.3° ± 2; *p* = 0.76) (Figure 5). The joint line orientation was corrected after the TKA implantation. The outliers for the HKA angle and the MPTA had a preoperative HKA angle inferior to 170° and a preoperative MPTA inferior to 85°.

The mean differences between the planned angles and the measured values on postoperative radiographs were close to zero (Table 4).

### 3.2. Patellar Tracking

The results of the patellar tracking are summarized in Table 5. Only four patients had a patellar tilt superior to 5° postoperatively, with a cohort mean of 2.9° ± 3.2. The existence of significant patellar tilt preoperatively in the aforementioned four patients meant maintaining the anatomic variation in line with our alignment principles. There were significantly fewer patients with a patellar tilt postoperatively rather than preoperatively (Figure 2). There was no complication of the extensor mechanism.

### 3.3. Frontal Laxity

Surgical approach and osteophyte resection aside, no soft tissue release was required. No collateral release was required. Ligament balancing was obtained with adjustment of the tibial cut. The polyethylene thickness was either 10 or 11 mm for all patients. A total of 36 patients (80%) achieved the asymmetric flexion gap target (2−6 mm lateral laxity).

**Figure 6:**
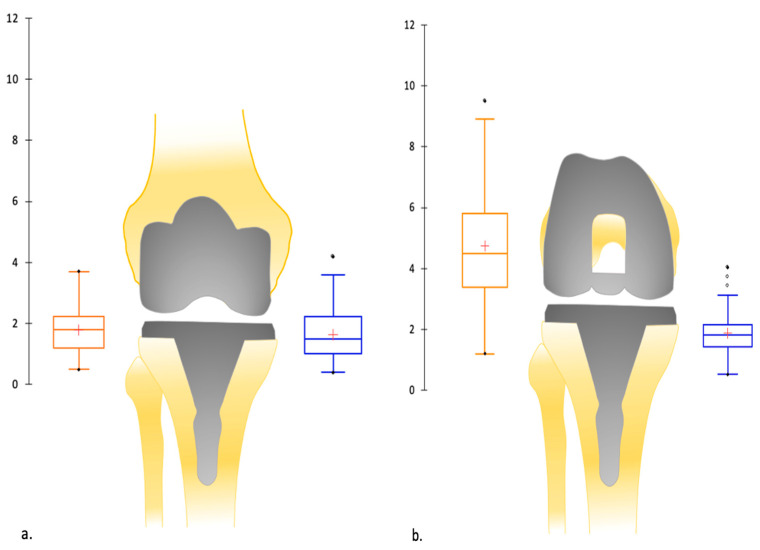
(**a**) Depiction by boxplots of the mean (in mm) of medial and lateral ligament balancing in extension after the TKA implantation: symmetric laxity in extension; (**b**) depiction by boxplots of the mean (in mm) of medial and lateral ligament balancing in flexion after the TKA implantation: asymmetric laxity in flexion. Symbols: cross indicates mean value; dot indicates outlier value.

No significant difference was evident between the medial gap laxity in extension, the medial gap laxity in flexion, and the lateral gap laxity in extension (Table 6, Figure 6). The lateral gap laxity in flexion was significantly higher than in extension or medial gap laxity, with a mean of +2.9 mm.

Nine patients (20%) did not have a higher lateral gap laxity in flexion but presented with a symmetric gap laxity in flexion. This is attributable to preservation of preexisting anatomic variation. No correlation has been found between this absence of lateral laxity in flexion and the preoperative or postoperative limb alignment (HKA angle, mMDFA, MPTA, slope) or the demographic data.

### 3.4. Clinical Improvement

The preoperative and postoperative Knee Society Scores along with a comparison of their difference is summarized in Table 7. There was marked improvement, as shown by differences pre- and postoperatively, in both the knee and function components of the score in all patients at the end of the follow-up period.

### 3.5. Complications

We are able to report only a single serious complication postoperatively and within the follow-up period. A female patient, in the early postoperative period, experienced delayed wound healing at a single tracking pin site with negative laboratory and culture tests. This required a revisit to the theatre on postoperative day forty-two for debridement and surgical closure. A second visit to the theatre was performed on postoperative day fifty-seven for further debridement and application of vacuum dressing. The aforementioned procedure was repeated on postoperative day 86. The patient was henceforth managed conservatively until resolution.

There were no revisions performed within this follow-up period.

## 4. Discussion

To personalize the knee alignment and hopefully improve patient satisfaction after TKA, different concepts of personalized alignment have been described these last years. An appropriate ligament balancing with an asymmetric gap in flexion also appears more physiological. Even so, this concept of asymmetric ligament balancing in flexion remains poorly researched. We hypothesized that a personalized alignment using AFIP has to restore the native knee alignment, keep a satisfying patellar tracking, and obtain physiological ligament balancing in extension (symmetric gap) and flexion (asymmetric gap). Therefore, we aimed to assess (1) the postoperative alignment of TKA, (2) the postoperative patellar tracking, and (3) the ligament balancing in extension and flexion after the TKA implantation. The results of this study show we were able to achieve our alignment goals in all patients, achieve patellar tracking, and achieve near physiologic ligament balancing in majority of the patients. We attribute the postoperative outliers in the three categories to our philosophy, in which complete eradication of pre-existing anatomic variation is not a feature. As such, our results are able to validate our hypothesis.

In a native non-osteoarthritic knee, the mean HKA angle can vary from 176.7° to 180.7° [5]. Personalized alignment strategies aim to restore this native non-osteoarthritic knee [12,14,15,16], with more or less restrictions on the tibial axis and/or on the femoral axis. Robotic assistance, owing to their accuracy, may make achieving non-neutral alignment targets more reproducible [43], diminishing the outliers’ risk in these personalized alignments, which keep some deformity. This robotic personalized alignment technique aimed to reduce the need for periarticular soft tissue releases and to restore the native knee kinematics, restoring the femoral driver of the knee. Our robotic-assisted technique allowed this femoral restoration without outliers. A common assumption is classically admitted that the outliers on the proximal tibia are more at risk of loosening or secondary displacement. A previous study on 398 knees found no difference in 15-year survivorship between TKA implants that were mechanically aligned (within 0° ± 3°) and those that were outside that range [44]. In spite of that, we remain circumspect about a tibial positioning with more than 5° of varus. Indeed, a finite element study reported that, for moderate (10°) and severe (15°) varus knee models, the maximum stress in kinematic alignment (KA) TKA increased by 24.8% and 32.2% compared with MA TKA [45]. Thus, a restricted KA for the tibial implant appears safer. In our study, the tibial axis was, as desired, partially restored with a moderate correction of the tibial varus. The decision to reproduce tibial slope within a cutoff limit, as evident by 68.9% falling within 3° of planned angles, is attributed to the use of a PCL-substituting implant in a geographical region where high slopes are prevalent. Notably, there were no differences in clinical outcome in these patients. The ROSA robotic system has already demonstrated its accuracy and reproducibility in previous cadaveric studies [46,47]. On 30 cadaveric knees, there were no significant differences between the planned and the measured resection values, except for femoral flexion that had a mean difference of -0.95°. In another cadaveric study comparing the accuracy of the ROSA Knee System with a conventional technique, the accuracy of bone resection angles was significantly improved for all values in the robotic group compared with the conventional group [47]. Our results confirmed the accuracy of this system.

In KA, the posterior femoral resection parallel to the posterior condylar axis [13] often results in a more internally rotated femoral component than in MA [48,49]. The femoral component would also be positioned 5° more in flexion in KA than in MA [50]. A recent study comparing 93 KA TKAs versus 93 MA TKAs reported a significantly higher incidence of lateral patellar tilt postoperatively in KA than MA [51]. Nevertheless, they also found a higher incidence of medial patellar tilt in MA, a sign of non-restoration of the native femoral rotation. In personalized alignment, using a femoral component designed for MA, and not KA, might lead to patellofemoral or flexion gap compromises [49,52]. The restoration of the mediolateral and radial locations of the groove and the sulcus angle of the native trochlea according to the alignment technique is heavily debated. Some studies affirmed that KA positioned the femoral implant with internal rotation increasing the risk of patellar maltracking [51,53,54]. Other studies reported an improvement in the patellar tracking due to KA, with a good restoration of the groove location and sulcus angle [48,49,52]. And the rotation of the femoral component, for its part, was not demonstrated as a strong factor of patellar maltracking [55,56]. It is difficult to consider all the parameters impacting the patellar tracking between native knee anatomy, MA TKA, or KA TKA [49]. Indeed, these different situations have an impact on the femoral rotation; the limb alignment and thus the Q-angle; the lateral location of the prosthetic groove according to the axis of the distal femoral cut; the flexion of the femoral component and thus the positioning of the lateral reach; etc. [48,52]. Currently, no unambiguous, robust study has shed light on the optimal alignment technique for patellar tracking. A good restoration of the gap laxity in flexion is primordial to achieve more physiological knee kinematics. But this goal should be performed without patellofemoral complication. Our results found a good patellar tracking in a population without preoperative patellar maltracking. Amongst our outliers for patellar tilt, attributed to preoperative anatomic variation, we report no differences in outcome. In our study, the implant was an anatomic design with a small increment of sizes, avoiding a significant flexion of the femoral component. A trochlea design adapted for the KA technique could improve the restoration of the native trochlea. Even so, our personalized alignment technique with this specific implant allowed us to restore the femoral anatomy without compromising the patellofemoral joint.

At the five-year follow-up period, all patients showed marked improvement in clinical and functional outcome, as indicated by difference between Knee Society Scores preoperatively compared with scores at last follow-up. The mean final KSS score being over 90 is also indicative of excellent clinical outcome, suggesting that this technique is capable of restoring near-normal functionality and improving overall quality of life. Given the reported single complication, diligent attention must be paid to the healing of surrounding soft tissue apart from the incision site.

The literature is unclear regarding the improvement in functional outcomes with personalized alignment compared with MA, and it remains extensively debated [14,33,50,57,58,59,60,61,62]. This uncertainty has been attributed to a lack of statistical power or non-discriminating functional scores. Despite that, the targets for knee restoration are probably not completely exact. Indeed, for most personalized alignment techniques reported in the literature, the ligament balancing is described as symmetrical in extension and flexion. This assumption is classically admitted despite the fact that, in native knees, there is a higher lateral laxity in flexion than in extension or than in the medial compartment [18,20,21]. The restoration of native knee kinematics necessitates the restoration of the ligament balancing in extension and flexion and thus an essentially asymmetric gap in flexion (+2 to 6 mm in the lateral compartment). In our study, the restoration of the femoral anatomy and a partial correction of the tibial varus enabled us to achieve this ligament balancing (symmetric gap in extension and asymmetric gap in flexion) in 80% of cases. For the patients who did not meet the asymmetric flexion target, we attribute the precedence of anatomic features during preoperative planning, respecting the soft tissue envelope and maintaining joint line obliquity—in line with our alignment philosophy. The symmetric posterior femoral resection avoids closing the lateral compartment in flexion (as in MA with the lateral rotation). During the planning, the preoperative values of knee laxity recorded with the robotic-assisted system should be interpreted cautiously. Indeed, the wear laxity, the collateral ligaments tightness due to osteophytes, and the presence of cruciate ligaments modify the physiological knee laxity. Hence, a rigorous surgical technique with precise planning for the bone resections was primordial in this personalized alignment technique. In the literature, lateral femoral condylar lift-off in flexion was reported and described as an inadequate and not fully understood ligament balancing [63,64]. This effect seemed more important for TKA without conservation of cruciate ligaments. Indeed, in vitro studies reported a higher lateral laxity in flexion after the PCL resection [65,66]. However, when the PCL is resected with concurrent conservation of the medial soft tissue in posterior stabilized TKA, akin to the technique of using the MCL landmark [32], the increase in flexion gap is shown to be less than 1 mm [67]. It is also worth noting that the PCL is not completely spared during CR TKA, with studies showing significant portions being removed [68,69]. Targeting a symmetric gap in flexion within the conventional technique arose from the fear of instability in flexion with posterior stabilized TKA. Furthermore, it remains to be proven that preoperative gaps can be accurately used to determine the final resection target. Notwithstanding, with the robotic-assisted system, the accuracy of the implants’ positioning and the possibility to check the ligament balancing at the end of the procedure avoid a significant risk of instability. In our study, the target of ligament balancing was obtained with measured resection in most of TKA, without release or thick polyethylene. Only one clinical study on cruciate-retaining TKA performed by kinematic alignment has assessed the clinical outcomes according to the lateral laxity in flexion [22]. They reported significantly better scores for clinical outcomes in knees with lateral flexion laxity greater than 2 mm.

Several limitations should be outlined in our study. Firstly, the mean follow-up was short—a longer time frame, approaching ten years or more, is necessary to observe outcomes past the mid-term. Nonetheless, this study aimed to assess the three postoperative parameters determining a satisfying restoration of the native knee biomechanics (the native alignment, the patellar tracking, and the gap laxity) along with clinical outcome and complication rate. Secondly, a small number of patients were observed. In addition, no control group was used. Although a comparative study at 1 year with conventional TKA showed no difference in outcomes [26], another case-controlled study is imperative to compare these results with the conventional technique. Nonetheless, we wanted to quickly evaluate and report the preliminary round of results in order to modify this technique early if needed. Third, the gap laxity was assessed only during the surgery by the robotic-assisted system. Notably so, very few existing systems are capable of reliably evaluating gap laxity in vivo. And although gap laxities were recorded with a clinically relevant and consistent technique, the forces involved were not quantified; here lies potential for future study using quantitative measurements. Furthermore, only varus knees were within the scope of this series, and future studies will have to evaluate the efficacy and safety of this system in valgus knees. It is known that valgus deformities can prove to be relatively challenging cases [27]. Analysis of cost for this system, which requires a different study design, has been conducted elsewhere in the literature and was not an aim of this study. This study was designed to be mono-centric and single-surgeon.

Future pathways of study must include prospective comparison against conventional TKA. To comprehensively assess implant survivorship, patients’ functional outcomes, and quality of life, posterior-stabilized TKA with an asymmetric gap in flexion should be evaluated against conventional TKA in the long term by the 10-year mark. Additionally, studies in valgus knees are required to determine generalizability across knee phenotypes.

## 5. Conclusions

For varus knees, this robotic-assisted system using a personalized alignment technique (kinematic femur, hybrid tibia) with anatomo-functional implant positioning allowed restoration of native knee alignment with a “safe zone” [70] for the tibial implant, keeping satisfying patellar tracking and restoring asymmetrical gap laxity in flexion with a higher laxity in the lateral compartment in majority of patients, thereby validating our hypothesis. Along with favorable increases in clinical scores, we are also able to report no major complications or revisions during this time period. To date, this is the longest survivorship reported for this system.

Limitations of this study include a lack of a control group, the follow-up period being too short to assess long-term outcomes, and the exclusion of valgus knees. The results of this series at five years were shown to be encouraging; however, further case-controlled studies against conventional TKAs are necessary. Going forward, studies on valgus knees, studies with 10-year follow-up, and the use of quantitative measures of gap laxity are required.

## Figures and Tables

**Table 1:** Preoperative demographics and clinical data.

	Mean ± SD	[Min; Max]
Age (years)	60.8 ± 7.5	[41; 80]
BMI (kg/m^2^)	33.5 ± 5.4	[23.2; 45.9]
Side (right)	24 (53.3%)	
Gender (male)	16 (35.6%)	
Flexion	115 ± 6.9	[100; 120]
Flexion contracture	8.5 ± 5.3	[0; 20]

BMI: Body mass index; SD: standard deviation.

**Table 2:** Intra-rater reliability and intraclass correlation coefficients.

Measurement Name	N (Radiographs/Cases)	ICC_3,1_ Value	95% Confidence Interval	Interpretation
HKA Angle	45	0.95	(0.92–0.97)	Excellent
mMPTA	45	0.92	(0.88–0.95)	Excellent
mMDFA	45	0.93	(0.89–0.96)	Excellent
Tibial Slope	45	0.88	(0.81–0.93)	Good
Posterior Offset	45	0.77	(0.66–0.85)	Good
JLO	45	0.82	(0.72–0.89)	Good
Medial Gap in Extension	45	0.94	(0.90–0.96)	Excellent
Lateral Gap in Extension	45	0.91	(0.86–0.94)	Excellent
Medial Gap at 90°	45	0.89	(0.82–0.94)	Good
Lateral Gap at 90°	45	0.87	(0.79–0.92)	Good

**Table 3:** Preoperative and postoperative radiographic measurements and outliers.

	Preoperative Data	Postoperative Data	*p* Value
HKA (°) (mean ± SD) [Min; Max]	170.2 ± 3.9 [163.5; 179]	177.4 ± 2.2 [172; 182]	<0.0001
OUTLIERS: HKA < 175°	-	4 (8.9%)	-
mMDFA (°) (mean ± SD) [Min; Max]	91.3 ± 2 [87; 94]	91.1 ± 1.5 [88; 94]	0.76
OUTLIERS: mMDFA > 95°	-	0	-
OUTLIERS: mMDFA > 93°	-	1 (2.2%)	-
MPTA (°) (mean ± SD) [Min; Max]	85.7 ± 2.1 [82; 90]	87.4 ± 1.8 [83; 90]	<0.0001
OUTLIERS: MPTA < 85°	-	2 (4.4%)	-
OUTLIERS: MPTA < 87°	-	11 (24.4%)	-
JLCA (°) (mean ± SD) [Min; Max]	6.2 ± 2.2 [1; 12]	-	-
JLO (°) (mean ± SD) [Min; Max]	−0.9 ± 2.4 [−7; 5]	0.9 ± 1.7 [−2; 4]	0.0002
OUTLIERS JLO > 3°	-	3 (6.7%)	-
Slope (°) (mean ± SD) [Min; Max]	81.7 ± 3.1 [74; 89]	86.8 ± 1.8 [83; 91]	<0.0001
OUTLIERS Slope < 80°	-	0	-
Difference Post–Pre of Posterior Femoral offset (mm) (mean ± SD) [Min; Max]	1.9 ± 1 [0.09; 5.4]		-

HKA: Hip knee ankle angle; mMDFA: mechanical medial distal femoral angle; MPTA: medial proximal tibial angle; JLCA: joint line convergence angle; JLO: joint line orientation; SD: standard deviation.

**Table 4:** Difference between the planned angles and the radiographic angles measured postoperatively.

	Mean ± SD	[Min; Max]	% Within 3°	% Within 2°
mMDFA	−0.2 ± 1.5	[−3.2; 3.8]	95.6%	86.7%
MPTA	−1 ± 1.7	[−4; 3]	93.3%	80%
Tibial slope	3.5 ± 1.9	[0; 8]	68.9%	55.6%

**Table 5:** Radiographic measurements of the patellar tracking before and after TKA.

	Preoperative Data	Postoperative Data	*p* Value
Patellar tilt (°) (mean ± SD) [Min; Max]	4.4 ± 4.5 [0; 14]	2.9 ± 3.2 [0; 11]	0.146
Patellar tilt > 5°	14 (31%)	4 (8.9%)	0.016
Patellar translation (mm) (mean ± SD) [Min; Max]	0.6 ± 2.4 [0; 14]	0.4 ± 1.4 [0; 6]	0.763
Patellar translation > 5 mm	2 (2.4%)	1 (2.2%)	1.000

SD: Standard deviation.

**Table 6:** Ligament balancing in the medial and lateral compartments in extension and flexion, measured at the end of the TKA procedure.

Frontal Laxity (mm) (Mean ± SD) [Min; Max]	Medial	Lateral	*p* Value
Extension	1.7 ± 0.9 [0.5; 4.3]	1.8 ± 0.8 [0.5; 3.7]	0.7900
Flexion at 90°	1.9 ± 0.8 [0.5; 4]	4.7 ± 2 [1.2; 9.5]	<0.0001
*p* value	0.54	<0.0001	-
Gap in flexion—Gap in extension	0.1 ± 1.1 [−2.9; 2.3]	2.9 ± 2.2 [−2.4; 7.2]	0.0064

SD: Standard deviation.

**Table 7:** Knee Society Scores calculated preoperatively and at last follow-up with their difference.

	Mean ± SD	[Min; Max]
Preoperative score		
KSS score—knee	38.3 ± 7.6	[25; 65]
KSS score—function	39.8 ± 13.1	[0; 60]
Score at last follow-up		
KSS score—knee	94.1 ± 8.5	[60; 100]
KSS score—function	91.6 ± 10.9	[60; 100]
Difference between preoperative and follow-up scores		
KSS score—knee	55.86 ± 11.5	[19; 71]
KSS score—function	51.84 ± 15.3	[10; 90]

KSS: Knee Society Score; SD: standard deviation.

## Data Availability

Data for this study is held within the electronic medical records at the International Knee and Joint Centre—Abu Dhabi, UAE. Restrictions apply to the datasets. The datasets presented in this article are not readily available due to regulations concerning patient confidentiality stipulated by the Department of Health—Abu Dhabi, United Arab Emirates. Requests to access the datasets should be directed to the corresponding author and/or International Knee and Joint Centre—Abu Dhabi, UAE.

## Abbreviations

The following abbreviations are used in this manuscript:

TKA	Total knee arthroplasty
HKA	Hip knee ankle
MPTA	Medial proximal tibia angle
mMDFA	Medial distal femoral angle
JLCA	Joint line convergence angle
JLO	Joint line orientation
KA	Kinematic alignment
PA	Personalized alignment
AFIP	Anatomo-functional implant positioning
MCL	Medial collateral ligament
PCL	Posterior cruciate ligament
